# Does supine versus lateral position affect operative time and reduction quality for elderly intertrochanteric femur fractures?

**DOI:** 10.1007/s00590-026-04739-5

**Published:** 2026-04-15

**Authors:** Ryan C. Ross, Soroush Shabani, Brandan Sakka, Jorge Figueras, Michael Allen, Jackson Lee, Charalampos Zalavras, Lane Shepherd, Joseph T. Patterson, Joshua L. Gary

**Affiliations:** 1 Department of Orthopaedic Surgery, University of Southern California, Los Angeles, USA; 2 Department of Orthopaedic Surgery, University of California, San Francisco in Fresno, Fresno, USA

**Keywords:** Intertrochanteric fracture, Fragility fracture, Positioning, Hip fracture

## Abstract

**Purpose:**

To compare operative time, reduction quality, and implant placement between supine and lateral decubitus positions for cephalomedullary nailing (CMN) of intertrochanteric fractures in elderly patients.

**Methods:**

A retrospective review was conducted of patients aged ≥ 60 years with intertrochanteric fractures treated with CMN at a Level I trauma center over one year. Patient demographics, injury characteristics, and operative details were recorded. Procedures were performed on either a traction table or flat-top table. The primary outcome was total operating room (OR) time. Secondary outcomes included setup time, surgical time, reduction grade, tip-apex distance, implant placement, blood loss, fluoroscopic exposure time, fluoroscopic radiation dose, and postoperative complications.

**Results:**

54 patients positioned supine and 29 positioned lateral decubitus were included. No differences were observed between groups regarding patient and injury characteristics, including operating table type (*p* > 0.05). Lateral group, compared to supine group, had significantly greater total OR time (218.2 ± 60.8 vs. 162.8 ± 45.4 min, *p* < 0.001), setup time (90.9 ± 21.5 vs. 64.1 ± 21.2 min, *p* < 0.001), surgical time (114.5 ± 43.4 vs. 85.8 ± 33.4 min, *p* < 0.001), and fluoroscopic radiation dose (13.5 ± 13.8 vs. 6.1 ± 3.7 Gy·cm^2^, *p* < 0.001). There was no significant difference in reduction grade, implant placement measures, blood loss, fluoroscopy time, or postoperative complications (*p* > 0.05). On multivariate regression, lateral positioning was significantly associated with increased total OR time (β = 42.17, *p* = 0.004), setup time (β = 21.55, *p* < 0.001), surgical time (β = 21.18, *p* = 0.040), and radiation dose (β = 6.37, *p* = 0.009).

**Conclusion:**

Supine positioning for CMN of intertrochanteric fractures in elderly patients was associated with shorter operative time compared to lateral positioning, with no difference in reduction quality or implant placement.

## Introduction

Hip fractures are a common injury in the elderly population, with an incidence that is predicted to double from 2018 to 2050 [1]. Intertrochanteric femur fractures account for approximately half of all hip fractures [2, 3]. These injuries are associated with older age, more comorbid medical conditions, longer hospital stays, worse functional recovery, and higher mortality compared to femoral neck fractures [2–4].

The standard-of-care management of intertrochanteric fractures in ambulatory patients is surgical fixation to allow for early mobilization and weight bearing. Stable fracture patterns (AO Foundation/Orthopedic Trauma Association (AO/OTA) classification 31A1) can successfully be treated with a sliding hip screw or cephalomedullary nail (CMN), while reverse obliquity and unstable patterns (AO/OTA 31A2 and 31A3) are commonly treated with CMN after reduction [5]. Recent treatment trends have shown a surgeon preference for CMN fixation for all intertrochanteric fracture patterns [6, 7]. Regardless of the chosen implant, accurate reduction and ideal implant placement are crucial to good outcomes.

Reduction and fixation of intertrochanteric fractures can be performed in a variety of patient positions. The most common positions include supine or lateral decubitus, with or without on-table limb traction. Each position confers various advantages and disadvantages related to ease of setup, maintenance of reduction, and fluoroscopic image acquisition [8, 9]. Several studies have compared the commonly preferred technique of supine position on a traction table with lateral decubitus position on a radiolucent flat-top table. Others have included supine and semilithotomy positions on a radiolucent table. There is limited literature that discusses lateral position on a traction table. There have been mixed conclusions in the literature regarding setup time, surgical time, reduction quality, and tip-apex distance (TAD) [10–12].

As much of the elderly population at risk of intertrochanteric fractures has comorbidities and heightened vulnerability to prolonged surgery, minimizing operative time without compromising reduction quality is beneficial [13]. This study was conducted to determine if there is a difference in operative time between supine and lateral positions for intertrochanteric fracture fixation in elderly patients. We hypothesized that supine positioning is associated with shorter total operating room (OR) time, with no difference in reduction quality or implant placement.

## Methods

A retrospective cohort study was conducted at one Level 1 trauma center in the United States. All patients with proximal femur fractures involving the trochanteric region from January 2022 to December 2023 were identified from hospital case logs. Patients ≥ 60 years of age with intertrochanteric fracture patterns classified as AO/OTA 31A1, 31A2, or 31A3 who were treated operatively with CMN were included. Intertrochanteric fractures with subtrochanteric extension were included, while subtrochanteric and periprosthetic fractures were excluded. Patients without saved intraoperative fluoroscopic images and those who underwent concomitant surgical procedures in addition to intertrochanteric fracture fixation were excluded. An interquartile range analysis of mean total OR time by surgeon was used to identify outliers.

Medical records were retrospectively reviewed to collect patient demographics (i.e. age, sex, body mass index (BMI)), injury details (i.e. mechanism, laterality, AO/OTA class), operative details (i.e. American Society of Anesthesiologists classification (ASA), patient position, traction use and method, reduction method, implant characteristics, operative time, fluoroscopic exposure), and clinical outcomes, including postoperative complications and readmission within 30 days. Postoperative complications were defined as surgical or medical adverse events occurring during the index hospitalization following surgery.

Operating table and intraoperative traction method was recorded as traction table, radiolucent flat-top table with manual traction, or radiolucent flat-top table with skeletal traction. Traction pin use was recorded as no traction pin used, preoperative skeletal traction removed, or traction pin placed and removed during the operation. Reduction method was recorded as closed, open, or percutaneous. CMN length was classified as short or long, and CMN proximal fixation was with either a helical blade or lag screw. Blood loss was calculated using a modified Mercuriali formula with preoperative hematocrit, postoperative nadir hematocrit, perioperative blood transfusions, and blood volume estimated using the Nadler formula [14].

Intraoperative fluoroscopic images were assessed to determine AO/OTA classification of each fracture. Intraoperative images were analyzed with measurement tools in the institution’s picture archiving and communication system (PACS) software to determine reduction grade according to the Baumgaertner criteria (poor, acceptable, good) and TAD [15]. Anterior–posterior (AP) thirds and lateral (LAT) thirds analysis was conducted as described by Peters et al. to determine if the CMN helical blade or lag screw resided within the superior, middle, or inferior third of the femoral head on AP images and anterior, middle, or posterior third on LAT images [12]. Blades and screws touching the borderline between the thirds sections were recorded as superior-middle or inferior-middle on AP images and anterior-middle or posterior-middle on LAT images as appropriate. Imaging analysis was conducted by four authors, including two orthopaedic surgery residents and an orthopaedic trauma fellow. Reviewers independently assessed a subset of patients and were not blinded to patient group due to the retrospective design and use of institutional PACS.

The primary outcome was total OR time, defined as the time from patient entry of the OR to exit. Secondary outcomes included setup time (time from OR entry to first incision), surgical time (time from incision to skin closure), Baumgaertner reduction grade, TAD, AP Thirds placement, LAT Thirds placement, blood loss, fluoroscopy exposure time, fluoroscopy dose, postoperative complications, and 30-day readmission.

Wilcoxon rank-sum and chi-square analyses were used to assess differences in baseline covariates and outcomes between position groups. Multivariable linear regression assessed associations between operative time parameters, TAD, blood loss, fluoroscopy time, and fluoroscopy dose with position groups. Multivariable logistic regression analyzed associations between postoperative complications and position groups. Multivariable ordinal regression evaluated the relationship between Baumgaertner grade, AP Thirds placement, and LAT Thirds placement with position groups. The multivariable regressions considered patient demographics, injury characteristics, and operative details. Covariate selection was optimized through Lasso regression with cross-validation to identify the optimal lambda value. The final model included covariates of position, age, BMI, ASA, traction pin use, reduction method, and CMN proximal fixation. A *p*-value < 0.05 was considered significant. Missing values were not imputed. Statistical analyses were performed with R (version 4.4.1, R Foundation, Vienna, Austria).

## Results

**Fig. 1:**
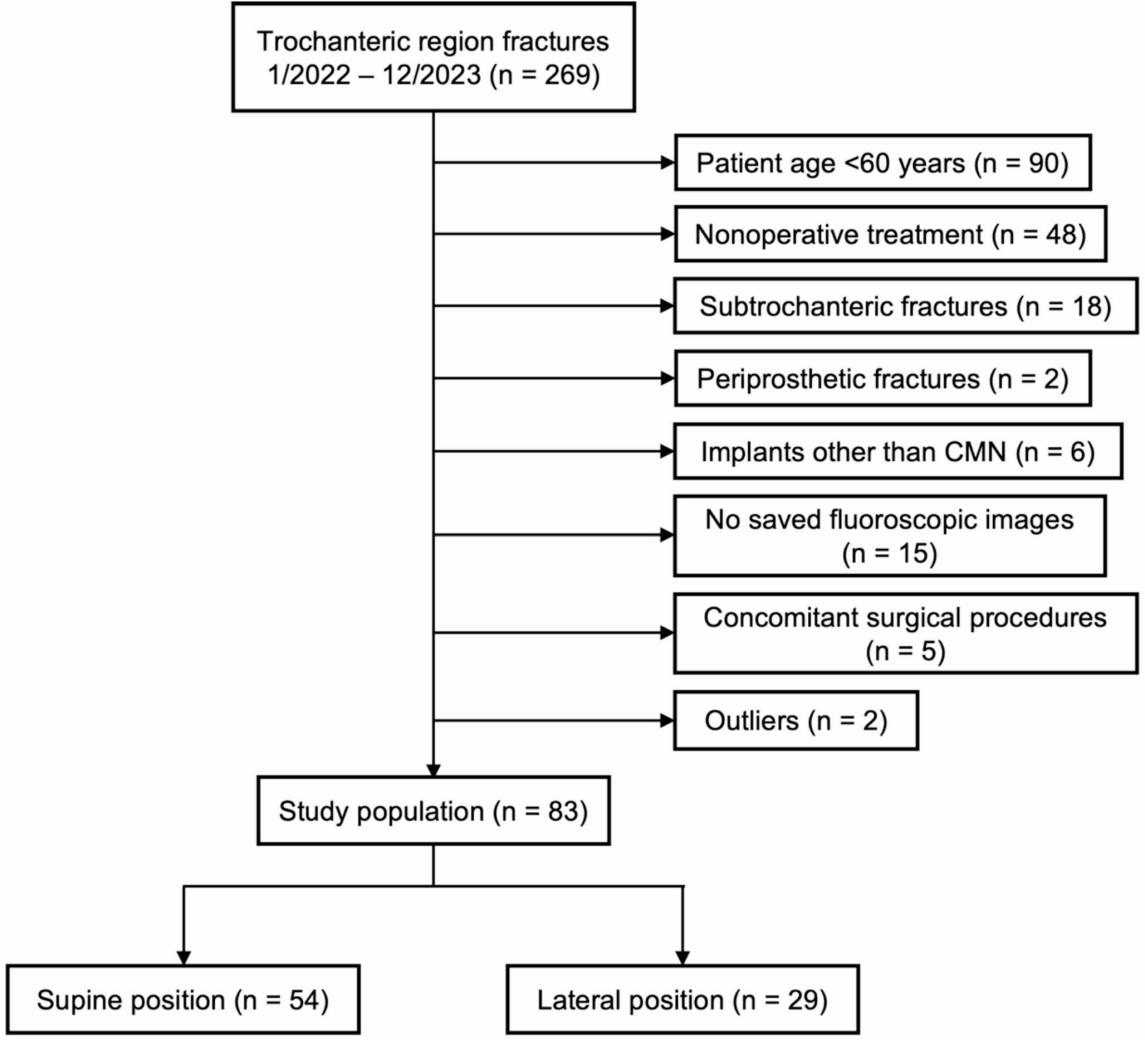
Flowchart of patient selection

A total of 269 patients with trochanteric region femur fractures were identified and screened. After application of exclusion criteria, one patient was excluded as an outlier based on the interquartile range analysis described above. An additional patient was excluded due to a peri-induction complication resulting in hemodynamic instability and a three-hour delay between anesthesia induction and the start of surgery. In sum, 186 patients were excluded (Fig. 1). The study cohort included 83 patients with intertrochanteric fractures: 54 in supine position (65.1%) and 29 in lateral position (34.9%). The average age was 79.8 ± 10.5 years, and the average BMI was 24.3 ± 5.0 kg/m^2^. 57 patients were female (68.7%), and ASA class 3 was most common in both groups. There was no significant difference in age, sex, BMI, or ASA between supine and lateral patients (Table 1).

**Table 1:** Demographics and intraoperative measures

Term	Total *N* = 83	Supine position *N* = 54	Lateral position *N* = 29	*p*-value
Age	79.8 ± 10.5	80.3 ± 10.5	78.9 ± 10.5	0.603
BMI (kg/m^2^)	24.3 ± 5.0	24.1 ± 5.0	24.8 ± 5.0	0.459
Sex				0.231
Male	26 (31.3%)	14 (25.9%)	12 (41.4%)	
Female	57 (68.7%)	40 (74.1%)	17 (58.6%)	
ASA class				0.38
1	1 (1.2%)	1 (1.9%)	0 (0%)	
2	24 (28.9%)	18 (33.3%)	6 (20.7%)	
3	53 (63.9%)	31 (57.4%)	22 (75.9%)	
4	5 (6.0%)	4 (7.4%)	1 (3.4%)	
Laterality				0.42
Left	48 (57.8%)	29 (53.7%)	19 (65.5%)	
Right	35 (42.2%)	25 (46.3%)	10 (34.5%)	
AO/OTA class				0.794
31A1.2	11 (13.3%)	8 (14.8%)	3 (10.3%)	
31A1.3	33 (39.8%)	21 (38.9%)	12 (41.4%)	
31A2.2	8 (9.6%)	4 (7.4%)	4 (13.8%)	
31A2.3	21 (25.3%)	13 (24.1%)	8 (27.6%)	
31A3.1	1 (1.2%)	1 (1.9%)	0 (0%)	
31A3.3	9 (10.8%)	7 (13.0%)	2 (6.9%)	
Intraoperative measures				
Reduction method				0.21
Closed	63 (75.9%)	39 (72.2%)	24 (82.8%)	
Open	10 (12.0%)	6 (11.1%)	4 (13.8%)	
Percutaneous	10 (12.0%)	9 (16.7%)	1 (3.4%)	
Table type/traction method				0.089
Traction table	69 (83.1%)	42 (77.8%)	27 (93.1%)	
Flat-top table + manual traction	6 (7.2%)	4 (7.4%)	2 (6.9%)	
Flat-top table + skeletal traction	8 (9.6%)	8 (14.8%)	0 (0%)	
Traction pin				0.407
None	68 (81.9%)	42 (77.8%)	26 (89.7%)	
Removed only	5 (6.0%)	4 (7.4%)	1 (3.4%)	
Placed and removed	10 (12.0%)	8 (14.8%)	2 (6.9%)	
CMN proximal fixation				**< 0.001**
Helical blade	40 (48.2%)	36 (66.7%)	4 (13.8%)	
Lag screw	43 (51.8%)	18 (33.3%)	25 (86.2%)	
CMN length				0.114
Short	49 (59.0%)	28 (51.9%)	21 (72.4%)	
Long	34 (41.0%)	26 (48.1%)	8 (27.6%)	

Bolded values indicate statistical significance

BMI, body mass index; ASA, American Society Anesthesiologists; CMN, cephalomedullary nail

A significantly larger proportion of helical blades than lag screws were used for CMN proximal fixation in the supine group compared to the lateral group (36 (66.7%) versus 4 (13.8%), respectively, *p* < 0.001). Left-sided injuries, AO/OTA class of 31A1.3, closed reduction, traction table, no traction pin use, and short nail were the most common injury and operative parameters in both groups. No significant differences in laterality, AO/OTA class, reduction method, operating table/traction method, traction pin use, and nail length were observed between the groups (Table 1).

Total OR time was significantly longer in the lateral group than the supine group (218.2 ± 60.8 min vs. 162.8 ± 45.4 min, *p* < 0.001). Setup time was significantly longer in the lateral group than the supine group (90.9 ± 21.5 min vs. 64.1 ± 21.2 min, *p* < 0.001). Similarly, surgical time was significantly longer in the lateral group than the supine group (114.5 ± 43.4 min vs. 85.8 ± 33.4 min, *p* < 0.001) (Table 2). Baumgaertner reduction grade of “Good”, AP Thirds placement of “Superior Middle”, and LAT Thirds placement of “Middle” were most common in both groups. Baumgaertner grade of “Good” was observed in 50.0% of the supine group and 62.1% of the lateral group. Reduction and implant placement measures of Baumgaertner, TAD, AP Thirds, and LAT Thirds were not significantly different between position groups. Blood loss and fluoroscopy exposure time were not significantly different between the groups. However, the lateral group was exposed to a higher dose of fluoroscopy radiation than the supine group (13.5 ± 13.8 Gy·cm^2^ vs. 6.1 ± 3.7 Gy·cm^2^, *p* < 0.001) (Table 2).

**Table 2:** Comparison of operative outcomes across patient positioning

Term	Total *N* = 83	Supine position *N* = 54	Lateral position *N* = 29	*p*-value
Surgical time parameters				
Total OR time (min)	182.2 ± 57.5	162.8 ± 45.4	218.2 ± 60.8	**< 0.001**
Setup time (min)	73.5 ± 24.8	64.1 ± 21.2	90.9 ± 21.5	**< 0.001**
Surgical time (min)	95.8 ± 39.4	85.8 ± 33.4	114.5 ± 43.4	**< 0.001**
Reduction and implant placement measures				
Baumgaertner				0.52
Poor	5 (6.0%)	4 (7.4%)	1 (3.4%)	
Acceptable	33 (39.8%)	23 (42.6%)	10 (34.5%)	
Good	45 (54.2%)	27 (50.0%)	18 (62.1%)	
TAD (mm)	22.8 ± 6.6	22.4 ± 6.9	23.6 ± 5.9	0.453
AP Thirds				0.233
Superior	19 (22.9%)	11 (20.4%)	8 (27.6%)	
Superior-middle	42 (50.6%)	31 (57.4%)	11 (37.9%)	
Middle	22 (26.5%)	12 (22.2%)	10 (34.5%)	
Inferior-middle	0 (0%)	0 (0%)	0 (0%)	
Inferior	0 (0%)	0 (0%)	0 (0%)	
LAT Thirds				0.312
Anterior	0 (0%)	0 (0%)	0 (0%)	
Anterior-middle	12 (14.5%)	10 (18.5%)	2 (6.9%)	
Middle	67 (80.7%)	42 (77.8%)	25 (86.2%)	
Posterior-middle	4 (4.8%)	2 (3.7%)	2 (6.9%)	
Posterior	0 (0%)	0 (0%)	0 (0%)	
Intraoperative and postoperative measures				
Blood loss (mL)	315.4 ± 172.2	316.4 ± 160.2	313.6 ± 195.6	0.538
Fluoroscopy time (min)	2.1 ± 1.0	2.1 ± 0.9	2.3 ± 1.2	0.626
Fluoroscopy dose (Gy·cm^2^)	8.7 ± 9.3	6.1 ± 3.7	13.5 ± 13.8	**< 0.001**
Postoperative complications	16 (19.3%)	10 (18.5%)	6 (20.7%)	0.279
Readmission	5 (6.0%)	2 (3.7%)	3 (10.3%)	0.509

Bolded values indicate statistical significance

TAD, tip-apex distance; AP Thirds, anterior–posterior thirds placement; LAT Thirds, lateral thirds placement

Position did not significantly impact the frequency of postoperative complications (*p* = 0.279). Complications in the supine group included hematoma of the injured hip requiring bedside incision and drainage (*n* = 1), surgical site infection (*n* = 1), urinary retention (*n* = 2), acute kidney injury (*n* = 2), gastrointestinal bleed (*n* = 1), and three deaths in acutely ill patients with significant medical comorbidities. In the lateral group, complications included surgical site infection (*n* = 2), with one requiring irrigation and debridement, deep vein thrombosis (*n* = 1), catheter-associated urethral injury (*n* = 1), urinary tract infection (*n* = 1), and acute respiratory failure (*n* = 1). Two patients in the supine group were readmitted within 30 days compared to three patents in the lateral group (*p* = 0.509). Both supine patients experienced surgical site infections of their injured hip requiring irrigation and debridement. Readmissions in the lateral group included surgical site infection requiring irrigation and debridement (*n* = 1), respiratory failure (*n* = 1), and cardiac arrest (*n* = 1) (Table 2).

On multivariable regression, an increase in total OR time was associated with lateral positioning (β = 42.17, 95% confidence interval [CI] = 13.62–70.72, *p* = 0.004). Similarly, an increase in setup time was associated with lateral positioning (β = 21.55, CI = 9.56–33.55, *p* < 0.001). Additionally, an increase in surgical time was associated with lateral positioning (β = 21.18, CI = 1.00–41.36, *p* = 0.040) (Table 3). An increase in fluoroscopy dose exposure was also associated with lateral positioning (β = 6.37, CI = 1.65–11.08, *p* = 0.009). No significant association with position was found for Baumgaertner grade, TAD, AP Thirds, and LAT Thirds. Additionally, no significant association was found with blood loss, fluoroscopy time, and postoperative complications (Table 3).

**Table 3:** Multivariable regressions evaluating association of intraoperative and postoperative outcomes with lateral versus supine patient positioning

Term	β	Confidence interval	Standard error	*p*-value
*Linear regression*				
Total OR time	42.17	13.62–70.72	14.32	**0.004**
Setup time	21.55	9.56–33.55	6.01	**< 0.001**
Surgical time	21.18	1.00–41.36	10.12	**0.040**
TAD	0.981	− 2.87–4.84	1.93	0.613
Blood loss	5.16	− 81.77–92.08	43.59	0.906
Fluoroscopy time	0.434	− 0.19–1.06	0.31	0.170
Fluoroscopy dose	6.37	1.65–11.08	2.36	**0.009**

Term	Odds ratio	Confidence interval	Standard error	*p*-value
*Logistic regression*				
Postoperative complications	1.03	0.18–5.77	0.86	0.974
*Ordinal regression*				
Baumgaertner	1.64	0.49–5.60	0.62	0.420
AP thirds	2.55	0.82–8.43	0.59	0.113
LAT thirds	2.69	0.27–6.84	0.81	0.753

Bolded values indicate statistical significance

TAD, tip-apex distance; AP Thirds, anterior posterior thirds; LAT Thirds, lateral thirds

## Discussion

Supine positioning was more time-efficient than lateral positioning in this cohort of elderly patients undergoing CMN for intertrochanteric fractures. After adjustment for potential confounders, lateral positioning was associated with more than 42 min of additional total OR time compared to supine positioning. This difference was driven by longer setup and surgical times in the lateral group, each more than 21 min longer than the supine group. Some portion of the operative time difference may be attributable to the more frequent helical blade use in the supine group. However, CMN proximal fixation was included as a covariate in the multivariable model, and positioning remained independently associated with total OR time. The operative efficiency of supine positioning was not accompanied by significant differences in reduction quality, implant placement measures, or postoperative complications. Previous papers have described technical advantages to lateral positioning, including easier reduction and exposure [12, 16], but the operative time efficiency of supine positioning is an important clinical consideration.

Prior studies comparing the operative time of supine and lateral positions for intertrochanteric fracture fixation have shown mixed results. Two recent meta-analyses comparing supine on a traction table position versus lateral without a traction table reported the lateral group to have a significantly shorter setup time than the supine group. For skin-to-skin time, Daher et al. found no significant difference while Kwok and Nara described a trend toward shorter surgical time in the lateral group with standardized means analysis, though an unclear magnitude of this time difference [11, 16]. Of individual cohort studies, Peters et al. reported comparable setup time between supine and lateral positions, in contrast to other studies that found the lateral group to have significantly shorter setup time [7, 10, 12, 17]. Several of these studies have reported shorter surgical time with lateral positioning, whereas others found no difference. Two studies that included supine position on a radiolucent table versus lateral position on a radiolucent table had conflicting results regarding setup and surgical time [8, 18].

This variation in the literature in part reflects the usage of traction tables or radiolucent flat-top tables. The distribution of operating table and traction method (traction table, flat-top table without traction, or flat-top table with skeletal traction) was similar between our study cohorts. Thus, our findings reflect that supine positioning is more efficient, independent of traction table use. Supine positioning is often less technical for operating room staff and requires minimal equipment. The setup for lateral may take longer due to the need for more patient manipulation, padding, and placement of supports, including bean bags and chest rolls. Lateral positioning may also require more adjustments during setup and intraoperatively to aid with reduction and imaging, contributing to increased operative time.

The prolonged setup and surgical times associated with lateral positioning have important clinical implications as operative time is a well-established independent risk factor for complications, including surgical site infection [13, 19]. Further, lateral positioning increases patients’ exposure to the risks of prolonged anesthesia. This is particularly significant given the high prevalence of intertrochanteric fractures in older adults, who have reduced organ reserve, altered pharmacokinetics, and more comorbidities. This makes older adults more vulnerable to the adverse effects of anesthesia including cardiovascular instability, respiratory failure, delirium, and cognitive decline [20, 21]. Additionally, supine positioning offers more immediate and less obstructed access for resuscitative measures in the event of intraoperative complications.

Beyond patient safety, shorter operative time results in health system benefits of operating room efficiency and resource utilization. At high-volume trauma centers where optimized surgical throughput is imperative, shorter operative times translate to more cases and decreased costs. A 2018 study found the mean cost for one minute of OR time across California hospitals to be $37.45 in the inpatient setting [22]. Even a 40-min difference per case scales to substantial annual cost savings.

Importantly, the efficiency benefits of supine positioning were not accompanied by worse reduction or implant placement. Baumgaertner reduction grade, TAD, and screw/blade position as assessed by AP Thirds and LAT Thirds were comparable between groups in our study. These results are consistent with most prior work [7, 10, 11, 16]. However, Peters et al. reported that patients positioned laterally had a higher proportion of lag screws placed centrally in the femoral head [12]. Contrarily, Unal et al. found a greater predilection of the lag screw for the middle third on LAT Thirds analysis with the supine group [8]. The clinical significance of these findings is unclear. Some studies describe a mechanical advantage of lateral positioning in achieving and maintaining reduction due to the minimization of deforming forces and posterior sag of the distal fracture fragment [7, 8, 12]. In the present cohort, this theoretical advantage did not have a clinical impact.

Obtaining good-quality radiographs is crucial for successful CMN of intertrochanteric fractures in any position. Fluoroscopic imaging is challenging in the lateral position because the injured hip and contralateral hip are superimposed when obtaining a true lateral. Manipulation of the patient and/or C-arm is necessary to overcome this. There are mixed results in the literature of differences in fluoroscopy time and number of images between supine and lateral positions [10, 11, 16, 18]. In the current study, although fluoroscopy time was similar between groups, lateral positioning was associated with a higher radiation dose. This increases the risks associated with exposure to ionizing radiation for both patients and providers. Baratz et al. similarly found lateral position to be associated with greater radiation dose compared to supine for proximal femur fracture fixation [23]. They postulated this difference to be a result of lateral positioning requiring more utilization of fluoroscopic projections in the sagittal plane. This involves imaging through a larger volume of tissue and often more shots to confirm alignment [24, 25].

This study has several limitations. It includes a sample of patients at a single institution and is retrospective in nature, impacting generalizability and introducing risk of selection bias. Long-term outcomes, including fracture union and function, were not evaluated due to the limited availability of follow-up records. These outcomes depend on the quality of surgery performed. Lastly, our study included a relatively small sample size.

## Conclusion

Supine positioning for CMN of intertrochanteric fractures in elderly patients was associated with shorter operative time compared to lateral positioning, with no difference in reduction quality or implant placement.

## Data Availability

The datasets generated and analyzed for this study are not publicly available due to patient privacy and institutional restrictions, but are available from the corresponding author on reasonable request.
